# Interpretation of field potentials measured on a multi electrode array in pharmacological toxicity screening on primary and human pluripotent stem cell-derived cardiomyocytes

**DOI:** 10.1016/j.bbrc.2017.01.151

**Published:** 2018-03-18

**Authors:** L.G.J. Tertoolen, S.R. Braam, B.J. van Meer, R. Passier, C.L. Mummery

**Affiliations:** a Department Anatomy and Embryology, Leiden University Medical Centre, Einthovenweg 20, 2300 RC, Leiden, The Netherlands; b Pluriomics B.V., Biopartner Building 3, Galileiweg 8, 2333 BD, Leiden, The Netherlands; c Department of Applied Stem Cell Technologies, MIRA Institute for Biomedical Technology and Technical Medicine, University of Twente, 7500 AE, Enschede, The Netherlands

**Keywords:** Action potential, Field potential, Cardiomyocytes, Cardiotoxicity, Multi electrode array, Computational simulation

## Abstract

Multi electrode arrays (MEAs) are increasingly used to detect external field potentials in electrically active cells. Recently, in combination with cardiomyocytes derived from human (induced) pluripotent stem cells they have started to become a preferred tool to examine newly developed drugs for potential cardiac toxicity in pre-clinical safety pharmacology. The most important risk parameter is proarrhythmic activity in cardiomyocytes which can cause sudden cardiac death. Whilst MEAs can provide medium- to high- throughput noninvasive assay platform, the translation of a field potential to cardiac action potential (normally measured by low-throughput patch clamp) is complex so that accurate assessment of drug risk to the heart is in practice still challenging. To address this, we used computational simulation to study the theoretical relationship between aspects of the field potential and the underlying cardiac action potential. We then validated the model in both primary mouse- and human pluripotent (embryonic) stem cell-derived cardiomyocytes showing that field potentials measured in MEAs could be converted to action potentials that were essentially identical to those determined directly by electrophysiological patch clamp. The method significantly increased the amount of information that could be extracted from MEA measurements and thus combined the advantages of medium/high throughput with more informative readouts. We believe that this will benefit the analysis of drug toxicity screening of cardiomyocytes using in time and accuracy.

## Highlights

• Computational model for the translation of field potential to action potential.
• Validation of the model using patch clamp and multi electrode array.
• Quantification of I_NA_ modulation, prolongation and triangulation from MEA data.
• Validation of the correlations by measurements with known cardioactive drugs.

## 1 Introduction

Multi electrode arrays (MEAs) have been developed to measure electrical activity in neural and cardiac cells. They are now being increasingly used specifically to analyze pharmacological toxicity of newly developed or combinations of compounds on cardiomyocytes of the heart. Most recently, in combination with cardiomyocytes derived from human (induced) pluripotent stem cells (hiPSC-CMs) they have started to emerge as powerful tools to examine the ability of certain compounds to induce arrhythmias in the heart, which can lead to “Sudden Cardiac Death”. This represents a major toxic hazard for new drugs in pre-clinical evaluation [1]. In this context, the Food and Drug Administration recently initiated the “Comprehensive in Vitro Proarrhythmia Assay” which aims to establish robust methods to assess cardiac drug safety using hiPSC-CMs. Crucial will be the ability to measure electrical responses of the cardiomyocytes in an accurate and predictive yet medium- to high- throughput platform.

**Fig. 1:**
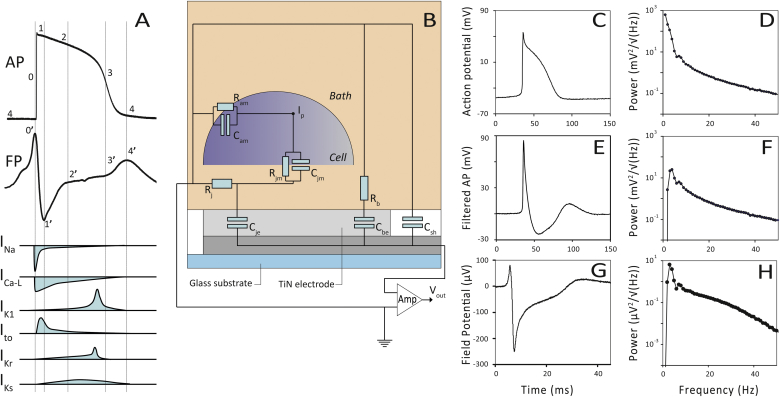
(A) A ventricular hPSC-CM AP and FP. Composing active currents are schematically depicted during time. (B) Equivalent circuit of a cell on a MEA. R_am_) apical cell membrane resistance to the bath; C_am_) apical membrane capacitance to the bath; C_jm_) cell membrane capacitance to junction; R_jm_) membrane resistance to the junction; R_b_) bath resistance; C_be_) electrode capacitance via the bath resistor; C_sh_) shunt capacitance of the electrode; R_j_) junction resistance; C_je_) junction capacitance of the electrode; I_p_) injection point of the AP (V_m_) in the simulation circuit. (C) Measured AP of a spontaneous beating mouse E17.5 cardiomyocyte. (D) Power spectrum of panel C, peak frequency at 1 Hz. (E) Result after Chybechev IIR filtering (F_c_ = 30 Hz) of the AP in panel C. (F) Power spectrum of panel E, peak frequency at ∼3.5 Hz. (G) Measured FP of a mouse E17.5 cardiomyocyte on a MEA. (H) Power spectrum of panel G, peak frequency at ∼3 Hz.

Classically, the cardiac action potential (AP) is measured using patch clamp electrophysiology in current clamp mode. All ionic currents of which the AP is composed can be measured individually in whole cell voltage clamp and have been studied in detail over several decades [2], [3], [4]. Alterations of one or more of these currents can lead to serious dysfunction of the heart, such as Torsade de Pointes (TdP) or prolongation of the “QT interval” on the standard electrocardiogram. Drug induced changes in the AP can be caused by modulations in Na^+^ and Ca^2+^ inward currents or several of the outward K^+^ currents, such as the rapidly activated (I_Kr_) and slow activated (I_Ks_) currents in human cardiomyocytes (Fig. 1A). Prolongation or shortening of repolarization can lead to concomitant modulation of the QT interval [5], [6], [7]. Parameters involved in determining the magnitude of these AP effects are the AP amplitude (APA; mV), the resting membrane potential (RMP; mV), the maximal rate of depolarization (V_max_; Vs^−1^) and AP duration at 50% and 90% of repolarization (APD_50_, APD_90_ respectively; ms). Although these parameters can be accurately measured using patch clamp electrophysiology, this is labour intensive and requires highly skilled experienced operators. By contrast, MEAs are much more user-friendly, are medium- to high- throughput in use but record the cardiac field potential (FP) instead of the AP. Prolongation or shortening of FP duration (FPD) are routinely measured on MEAs and are considered a measure of the APD_90_ [1], but other parameters are difficult to extract from FPs although they may actually contain a high level of information. In practice extraction of this information is hampered by poor knowledge of the underlying relationship (transfer function) between the AP and the FP. Here we present a robust basis for more informative MEA analysis by comparing simulated APs with their resulting FPs using an electrical circuit model. We validated our findings by analysis of FPs recorded on MEAs in hPSC-CMs exposed to drugs with known effects.

## 2 Material and methods

### 2.1 Patch clamp electrophysiology

Patch clamp electrophysiology was essentially done as described previously [8]. Microelectrodes with a resistance between 2 and 4 MOhm were made from Borosilicate glass (Warner Instruments, GC-150 T) with Flaming/Brown Micropipette Puller Model 97 (Sutter Instruments, CA). The sampling frequency was 5 kHz. During electrophysiological measurements, cells were kept in a buffer containing 145 mM NaCl, 5 mM KCl, 2 mM CaCl2, 2 mM MgCl2, 10 mM d-glucose and 10 mM HEPES, adjusted to pH 7.30 with NaOH. The pipette contained buffer consisting of 145 mM KCl, 5 mM NaCl, 2 mM CaCl_2_, 4 mM EGTA, 2 mM MgCl_2_, and 10 mM HEPES, adjusted to pH 7.30 with KOH.

### 2.2 Primary cardiomyocytes

Hearts of mouse embryos at embryonic day (E)17.5 were isolated by micro scalpel and washed in low calcium (Ca^2+^) medium for 30 min at room temperature. Tissue fragments were then incubated in enzyme-containing medium for 25–35 min at 37 °C. Dissociation of the tissue was completed in King's B medium by gentle shaking at room temperature for 1 h. The isolated cells were resuspended in Dulbecco's Modified Eagle Medium (DMEM) supplemented with 20% fetal calf serum (FCS) and incubated at 37 °C. MEA chips were plasma cleaned and coated with fibronectin (40 μg/mL) for 1 h at 37 °C. The isolated cardiomyocytes were allowed to attach to the surface of the MEA for 24 h in DMEM containing 5% FCS. Mouse experiments were performed conform EU Directive 2010/63/EU for animal experiments (Leiden University Medical Center).

### 2.3 hPSC-CM and differentiation

The human embryonic stem cell (hESC) line HES3 [9] was routinely cultured on 129SV mouse embryonic fibroblasts (MEFs) and induced to differentiate to cardiomyocytes as described previously [8]. Large numbers of contracting areas were obtained within 12 days that consisted of 20–25% cardiomyocytes.

### 2.4 MEA recordings

MEA chips were plasma-cleaned and coated with fibronectin (50 μg/mL) for 1 h at 37 °C. Clusters of beating hPSC-CMs were micro-dissected and replated on standard 60 electrode MEAs (Multi Channel Systems, Reutlingen, Germany). Extracellular recording was performed using a MEA1060INV MEA amplifier (Multi Channel Systems). Output signals were digitized at 10 kHz. Standard measurements were performed in DMEM supplemented with 5% FCS. During recordings, temperature was kept at 37 °C. Data were recorded using QT-screen (Multi Channel Systems) and analysed off-line with QT-analyser (Multi Channel Systems) or with LabVIEW software (see Supplemental Data). The MEAs used had titanium nitrate electrodes of 30 μm in size and a spacing of 200 μm. TTX was obtained from Alomone Labs (Jerusalem, Israel), Bay K 8644 from Tocris Bioscience (Bristol, United Kingdom).

### 2.5 Software and data analysis

Multisim (*Multisim 10.1.1* National Instruments, Austin, Texas, USA) SPICE based software was used for the transformation of APs to FPs according to the equivalent circuit depicted in Fig. 1B.

## 3 Results

### 3.1 Translation from AP to FP

A representative hPSC-CM AP measured by current clamp electrophysiology and a representative hPSC-CM FP measured by MEA is depicted in Fig. 1A. The important active currents in the different phases of the AP and FP are indicated.

The FP of a cell on a MEA electrode can be best understood from the electronic equivalent circuit (Fig. 1B). The main determinant of the circuit is the resistor-capacitance combination (R_j_-C_je_) resembling the junction resistance of the basal cell membrane and the MEA electrode. From a linear electronic circuit perspective it functions as a high pass filter. The filter characteristics of a single MEA electrode is modelled by applying a second order Chybechev digital infinite impulse response (IIR) filter with a half maximal cut off frequency (F_c_) of 30 Hz on an AP measured on a mouse E17.5 cardiomyocyte (Fig. 1E and C, respectively). Mouse cardiomyocytes were chosen for comparison of APs and FPs experiments because of their homogeneity and reproducibility in both electrophysiology and MEA measurements. Since the filter effect is the most dominant part of the basal cell membrane to electrode transfer function, we analysed frequency domains of both AP and FP signals by spectral analysis. Different values for F_c_ were applied (15–60 Hz) on the same AP. After the filter step, peak power frequencies were quantified (Table 1). The 30 Hz filtered example is depicted in Fig. 1E and F.

**Table 1:** Relationship between filter frequency in the time domain and the peak frequency in the frequency domain.

Filter frequency (Hz)	Peak power frequency of the filtered AP (Hz)
no filter	0.57
15	2.2
30	3.1
45	3.8
60	6.9

Next, the FP of mouse E17.5 cardiomyocytes was recorded with MEA (Fig. 1G) and spectrally analysed (Fig. 1H). This spectrum was almost identical to the power spectrum obtained from the filtered AP (Fig. 1F). Most FPs have a peak power frequency of around 3.5 Hz. This demonstrated that the transfer function of the MEA electrode can be modelled as a high pass filter with an F_c_ value around 30 Hz. The power spectrum of the unfiltered AP is shown in Fig. 1D.

### 3.2 Simulations

The underlying electrical transfer function of a MEA seems to act as a simple second order filter but in reality the final shape of the FP is dependent on the behaviour of all resistors and capacitors present in the circuit. Multisim was used (see Supplemental Data) to build the equivalent circuit depicted in Fig. 1B connected to a voltage source in order to inject the AP at node I_p_.

Membrane properties of ventricular mouse cardiomyocytes are well known from patch clamp electrophysiology (typically R_jm_ ∼ 500 MOhm and C_jm_ ∼ 30–70 pF). The contribution of the outer cell membrane resistance R_am_ and the capacity C_am_ on the final shape of the FP were negligible. Therefore, these components were not incorporated in our simulation model. Values for C_fb_ and C_sh_ were set to 100 pF. Different values for either capacitor did not affect the final shape of the FP but they could contribute to the overall noise of the output signal (results not shown). The value for the resistance of the bath (R_b_) was set to 500 Ohm Different substitutions for these parameters also had negligible effects on the shape of the resulting FP. Since the analysis of the frequency domain of MEA FPs revealed a F_c_ of 30 Hz, the resulting product of R_j_ and C_je_ is ∼5 F·Ohm (Eq. (1)).

(1) $$F_{c}=\frac{1}{2\pi R_{j}C_{je}}$$

**Fig. 2:**
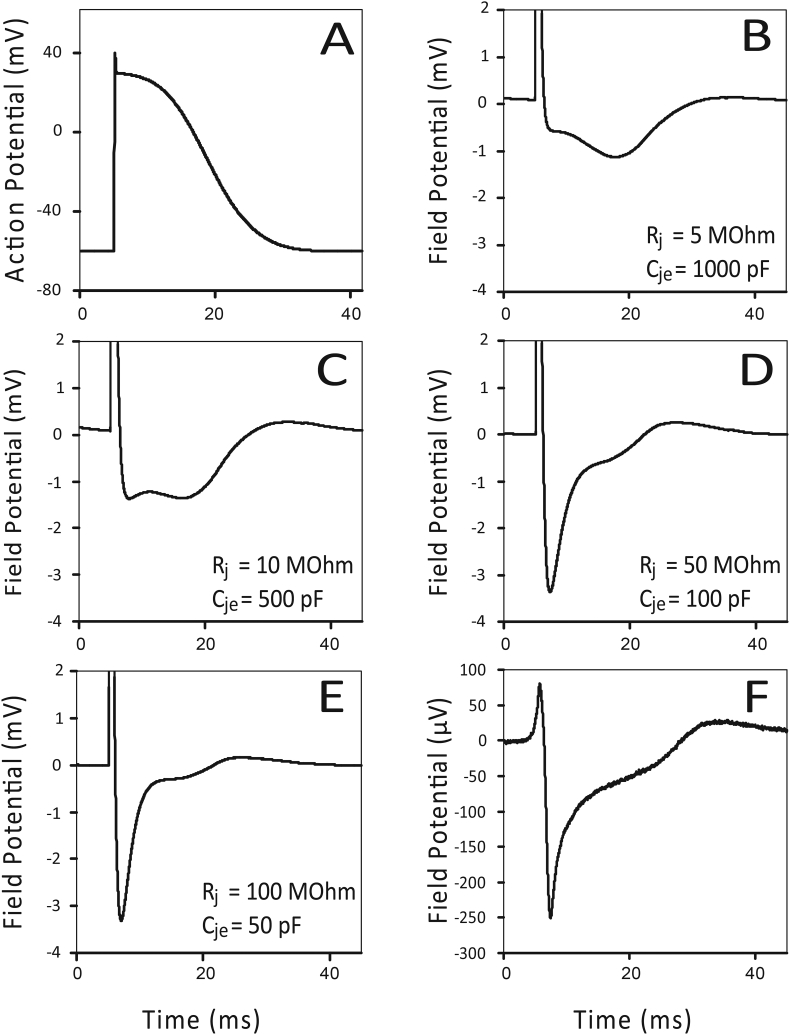
Simulated AP representative of a mouse E17.5 cardiomyocyte (A) and corresponding SPICE derived FPs with different values for R_j_ and C_je_ with a product constant 5000 pF·MOhm (F_c_ = 30 Hz) (B,C,D and E). Panels (D and E) result into the characteristic FP shape compared to a measured FP of a mouse cardiomyocyte E17.5 (F, repeated from Fig. 1G).

An mouse E17.5 cardiomyocyte AP was simulated (Fig. 2A) and the corresponding FPs were generated using different combinations for R_j_ and C_je_ with a product constant of 5 F·Ohm (Fig 2E and F; Table 2).

**Table 2:** The corresponding FPs are shown in Fig. 2B–E. Combinations with highest junctional resistances (R_j_ > 50 MOhm) are in good agreement with the measured FPs from embryonic mouse cardiomyocytes E17.5 on a MEA (Fig. 4F).

F_c_ (Hz)	R_j_ (MOhm)	C_je_ (pF)
30	5	1000
30	10	500
30	50	100
30	100	50

### 3.3 Modulation of the APs and FPs characteristic for hPSC-CMs

#### 3.3.1 Modulation of the inward Na^+^ current

**Fig. 3:**
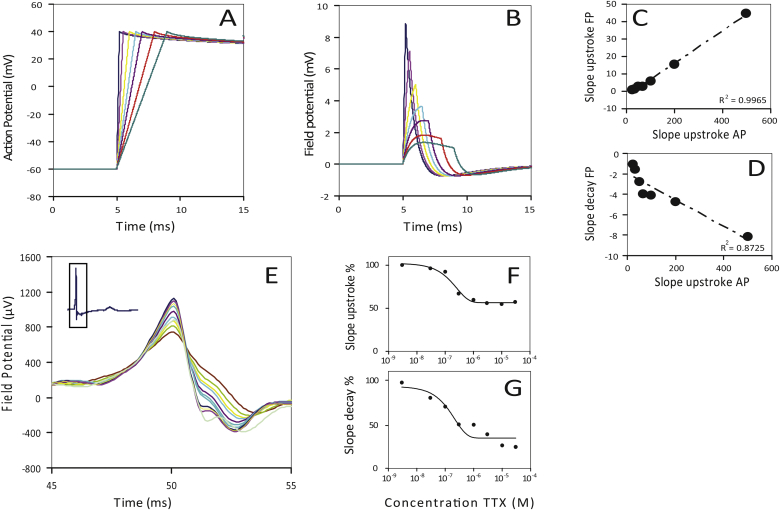
(A) Simulated APs of hPSC-CMs with different upstroke durations (0.2–4 ms) and (B) the corresponding SPICE derived FPs (C_je_ = 50 pF; R_j_ = 100 MOhm). Slope upstroke of the different APs against the slope upstroke (C) and slope decay (D) of the corresponding SPICE derived FPs. (E) FPs measured from hPSC-CMs, treated with different concentrations of tetrodotoxine (TTX). Slopes quantified from the upstroke (initiation to 0′) (F) and from the decay (0′ to 1′) (G).

APs of hPSC-CMs with different upstroke velocities (Na^+^ currents) were generated and analysed (Fig. 3A). The upstroke duration of the AP was varied from 0.25 to 4 ms resulting in V_max_ values of 400 Vs^−1^ to 25 Vs^−1^, respectively. To gain insight into the relationship between V_max_ of the AP versus the slope upstroke (from initiation to 0′ in Fig. 1A) and the slope decay (from 0′ to 1′ in Fig. 1A) of the FPs (Fig. 3B) they were plotted against each other and fitted by linear regression (Fig. 3C and D). Estimation of the slope upstroke and slope decay of the peak by linear regression was carried out over the trajectory from 45% to 95% of the peak or 45%–95% of the minimum amplitude of the FP.

**Fig. 4:**
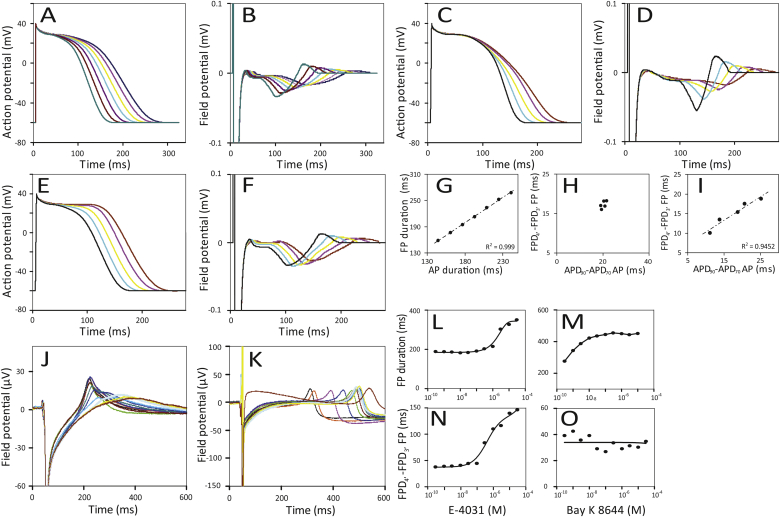
Simulated APs of hPSC-CMs with different durations (180–290 ms) (A) and corresponding SPICE derived FPs (B). The relationship of the APD versus the FPD is shown in panel G. Simulated APD variation due to K^+^ current modulation (C) and corresponding SPICE derived FPs (D). The ADP_90_-ADP_70_ values plotted versus the FDP_4**′**_-FDP_3**′**_ are shown in panel H. Simulated APD variation without K^+^ current modulation (E) and corresponding SPICE derived FPs (F). The ADP_90_-ADP_70_ values plotted versus the FDP_4**′**_-FDP_3**′**_ are shown in panel I. (J) FPs measured from hPSC-CMs exposed to E-4031. E-4031 evoked changes in K^+^ current and FPD prolongation (L) and modulation of the FDP_4**′**_-FDP_3**′**_ (N). Bay K 8644 (K) induced prolongation (M) without modulation of the FDP_4**′**_-FDP_3**′**_ (O).

In order to address the robustness of the relationship in practice, we investigated the effects for different values for C_je_ and R_j_ to model variation in cell attachment. Table 3 shows this behaviour. For the slope upstroke of the FP, the goodness of fit was relatively insensitive to different combinations of C_je_ and R_j_. However, the relationship between V_max_ and the slope decay was in general less linear (Fig. 3D).

**Table 3:** Linearity between V_max_ and slope upstroke and slope decay of the FP for different combinations of C_je_ and R_j ._

C_je_ = 50 pF R_j_ = 100 MOhm	R^2^
slope upstroke versus V_max_	0.9965
slope decay versus V_max_	0.8725

C_je_ = 100 pF R_j_ = 50 MOhm	R^2^
slope upstroke versus V_max_	0.9946
slope decay versus V_max_	0.7031

C_je_ = 500 pF R_j_ = 10 MOhm	R^2^
slope upstroke versus V_max_	0.9980
slope decay versus V_max_	0.8116

To compare the predicted behaviour of our simulations with actual MEA measurement data, hPSC-CMs were treated with increasing concentrations of tetrodotoxin (TTX) to block the sodium channels selectively. The peaks of the FPs are shown in Fig. 3E. Both slope upstrokes (Fig. 3F) and slope decays were analysed (Fig. 3G).

### 3.4 Modulation of the repolarizing phase of the AP

#### 3.4.1 APD prolongation, shortening and periodicity

APs were generated where the APD_90_ of the simulated hPSC-CM was varied over a range from 180 to 240 ms (Fig. 4A). FPs resulting from our model are shown in Fig. 4B. The FPDs were defined as the time between the peak of the upstroke (0′ in Fig. 1A) until the repolarization phase (4′ in Fig. 1A). The relationship between the APD and the FPD was assessed by linear regression (Fig. 4G). hPSC-CMs were then exposed to E-4031 (Fig. 4J) and Bay K 8644 (Fig. 4K) to shorten and prolong the FPD respectively, and measured by MEA. The dose response curves of the FPD prolongation for the compounds is shown in Fig. 4L and M.

Deviations from regular repetitive contraction, known as tachyarrhythmias or TdP, are visualized in Poincaré diagrams. They are accurately detected from current clamp recordings (APD and repetition interval). This method is also applicable to FPs (FPD and repetition interval) (Supplemental Fig. 2).

### 3.5 Modulation of the potassium currents during repolarization

**Supplementary Fig. 1:**
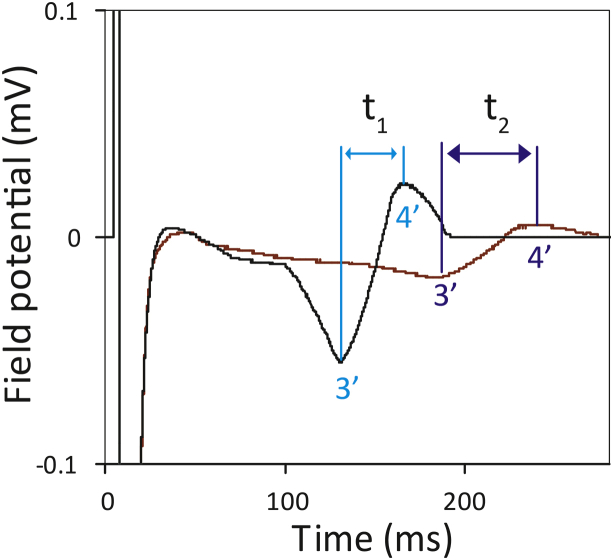
Method for quantification of triangulation of a FP by determining the distance between the peaks 3′ and 4′. Black trace: no drug (FPD = 170 ms); red trace: maximal prolongation with triangulation (FPD = 250 ms).

**Supplementary Fig. 2:**
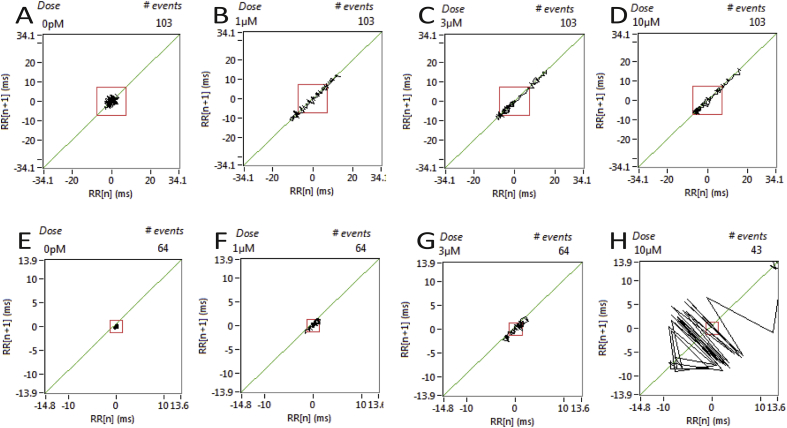
Poincaré diagrams from FPD at different drug concentrations (control, 1 μM, 3 μM and 10 μM) induced by (A) Bay K 8644 and (B) E-4031. The square indicates 10x the standard deviation from control.

Differences of APD_90_-APD_70_ are used to quantify modulation of I_Kr_, I_Ks_ currents. Triangulation, based on differences in APD_90_-APD_70_ (see Supplemental Data), is often used in cardiotoxicity assays [10]. Since the FP is strongly related to changes in the AP, we investigated whether it was possible to derive an indication of triangulation from FPs by measuring the interval of peak 3′ and 4′ (FPD_4**′**_-FPD_3**′**_; Suppl. Fig. 1). APs and corresponding FPs were generated that showed a high degree of triangulation (Fig. 4C and D, respectively). The relationship between the APD_90_ -APD_70_ and the FPD_4**′**_ -FPD_3**′**_ are in reasonably good agreement with a linear relationship (R^2^ = 0.9452) (Fig. 4I), while the APD_90_-APD_70_ and the FPD_4**′**_-FPD_3**′**_ of the prolonged APs and FPs without modulation of the potassium currents during repolarization (Fig. 4E and F, respectively) do not show triangulation.

To compare the predicted behaviour of our simulations with actual MEA data, FPs of hPSC-CMs were recorded on MEAs in the presence of E-4031 (Fig. 4J) and Bay K 8644 (Fig. 4K). E-4031, a class III antiarrhythmic agent, is well known for decreasing the I_Kr_ current, while Bay K 8644 induces FDP prolongation by increasing activation of the L-type Ca^2+^ channel. Dose response curves for triangulation (FPD_4**′**_-FPD_3**′**_) are shown (Fig. 4N and O). As expected, Bay K 8644 showed FPD prolongation (Fig. 4M) that was not accompanied by triangulation (Fig. 4O), while E-4031 showed both FPD prolongation (Fig. 4L) and triangulation (Fig. 4N) which is in line with our simulations.

## 4 Discussion

The AP is generated by a fast depolarization of the membrane potential due to Na^+^ channel activation. This ensures a strong inward current, activate the L-type Ca^2+^ channels and induces an increasing permeability to K^+^ as a result of the rapid activation of transient outward K^+^ channels (I_to_). In humans, this outward current, corresponding to negative change in membrane potential, allows activation of the rapid delayed rectifier (I_Kr_) and the slow rectifying (I_Ks_) K^+^ channels. Finally it leads to complete repolarization of the cardiomyocyte to its RMP. Commonly, consecutive changes of these currents can be deduced from changes in shape of the AP. Here we showed that many of these changes can be derived from the FP. As a direct result from our simulations, it is possible to interpret under various conditions which phenomenon underlies modulation induced by unknown drugs, using our simulation sets for typecasting. The interpretation and constraints in AP-FP translation are discussed below for different individual currents.

### 4.1 Sodium current modulation

V_max_ can be derived from both the slope upstroke and slope decay of the FP which can be obtained by linear regression. It is important to keep the trajectory over which the regression is computed well defined. In our study we used levels of 90%–45% of the peak amplitude. V_max_ can be obtained more accurately from the FP when extracted from the slope upstroke compared to the slope decay since the linearity is higher (respectively R^2^ = 0.9965 versus R^2^ = 0.8725).

### 4.2 FPD prolongation and shortening

Analysis of the FP duration is one of the most commonly used parameters in cardiac toxicity assays on MEAs. There is a good linear relationship (R^2^ = 0.999) between the AP duration and the duration of the resulting FP. We did not observe any deviation from linearity under varying conditions using different values of C_je_ and R_j_ with a constant product (F_c_ = 30 Hz, data not shown) modelling differences in cell attachment.

### 4.3 Modulation of the potassium currents during repolarization

In humans, drug-induced block of the rapid component of the K^+^ channels (I_Kr_ or I_Ks_) can induce ventricular tachyarrhythmias in the heart known as TdP. As a result, assays that measure I_Kr_ block have become standard tools for assessing cardiac hazard [11]. Here we show with simulations and known drugs that the presence of changes in APD_90_-APD_70_ measured from APs can be equally well detected and predicted by analysis of corresponding points from FPs (FPD_4**′**_-FPD_3**′**_).

In conclusion, to our knowledge we presented here the first comprehensive description of the cardiac FP in relation to cardiac toxicity screening and modulation of the underlying currents. We propose that it will improve the analysis of cardiac toxicity screening of cardiomyocytes using MEAs in general, but in particular of human *in vitro* models based on hPSC-CMs (hiPSC- or hESC-CMs). The approach we describe could serve as a framework for future medium- and high-throughput screening of new drugs and compounds for their potential harmful effects on the human heart.

## Funding

Work in the Mummery lab is supported by the European Research Council [grant number ERCAdG 323182 STEMCARDIOVASC].
