# Supplementary material for: Interpretation of field potentials measured on a multi electrode array in pharmacological toxicity screening on primary and human pluripotent stem cell-derived cardiomyocytes
Source: Biochem Biophys Res Commun. 2018 Mar 18;497(4):1135–41. doi: 10.1016/j.bbrc.2017.01.151 (PMC5854265; doi:10.1016/j.bbrc.2017.01.151)
Supplement: Supplementary file 1 [file mmc1.docx]

**Supplementary Material**

**Several programs modules were developed using LabVIEW (LabVIEW 8.5 and Multisim 11.0, National Instruments, Austin, Texas USA):**

- **Action Potential simulation module: generation of action potentials for Multisim input.**
- **A module for analysis of the simulated action potentials and field potentials (reading .lvm file format from Multisim).**
- **A module for analysis and overlay of action potentials and the resulting field potentials.**
- **A module for the analysis for the MEA recordings: Sodium slope, potassium modulation and APD/FPD duration analysis, power spectra.** Power spectra were generated using the Auto Power Spectrum module of LabVIEW. Linear regression was performed using the least bi-square method between 45% and 95% levels of the amplitude of the upstroke or decay phase of the field potential. Thresholds for amplitude and amplitude levels were incorporated in the software using the signal and measurement modules from LabVIEW***.***

For the source code of these programs contact: [L.G.J.Tertoolen@lumc.nl](mailto:L.G.J.Tertoolen@lumc.nl)

$\mathrm{Triangulation} = \frac{\left( {APD}_{90}-{APD}_{70} \right)_{\left[ drug \right]}-{({APD}_{90}-{APD}_{70})}_{[control]}}{{({APD}_{90}-{APD}_{70})}_{[control]}} \cdot100$ ( Eq. 1)

Supplementary Fig 1. Method for quantification of triangulation of a FP by determining the distance between the peaks 3’ and 4’. (1) Black trace: no drug (FPD=170 ms) and (2) Red trace: maximal prolongation with triangulation (FPD = 250 ms).

Supplementary Fig. 2. Poincaré diagrams from FP durations at different drug concentrations (control, 1 µM, 3 µM and 10 µM) *A*. Bay K 8644 induced. (*B*) E-4031 induced. The square indicates 10x the standard deviation from control.
